# ‘Pushing back’: People newly diagnosed with dementia and their experiences of the Covid‐19 pandemic restrictions in England

**DOI:** 10.1002/gps.5803

**Published:** 2022-09-02

**Authors:** Josie Dixon, Ben Hicks, Kate Gridley, Rotem Perach, Kate Baxter, Yvonne Birks, Carmen Colclough, Bryony Storey, Alice Russell, Anomita Karim, Eva Tipping, Sube Banerjee, DETERMIND team

**Affiliations:** ^1^ Care Policy and Evaluation Centre London School of Economics and Political Science London UK; ^2^ Brighton and Sussex Medical School University of Sussex Brighton UK; ^3^ Social Policy Research Unit University of York York UK; ^4^ School of Psychology University of Sussex Brighton UK; ^5^ Gateshead Health NHS Foundation Trust Gateshead UK; ^6^ Faculty of Health University of Plymouth Plymouth UK

**Keywords:** active social agent, active social citizenship, assets‐based, autonomy, coping, Covid‐19, dementia, pandemic, strengths‐based

## Abstract

**Background and Objectives:**

Research into people with dementia's experiences of the Covid‐19 pandemic has tended to focus on vulnerabilities and negative outcomes, with the risk of reproducing a discourse in which people with dementia are positioned as passive. Informed by concepts positioning people with dementia as *‘active social agents’*, we aimed to identify the pandemic‐related challenges faced by people recently diagnosed with dementia and examine the ways in which they actively coped with, and adapted to, these challenges.

**Research Design and Methods:**

In‐depth interviews with 21 people recently diagnosed with dementia, recruited through an existing national cohort. Data was analysed thematically using Framework.

**Findings:**

Key challenges included reduced social contact, loneliness and loss of social routines; difficulties accessing and trusting health services; dementia‐unfriendly practices; and disparate experiences of being able to ‘get out’ into the physical neighbourhood. People with dementia responded to challenges by maintaining and extending their social networks and making the most of ‘*nodding acquaintances*’; learning new skills, for communication and hobbies; supporting others, engaging in reciprocal exchange and valuing connection with peers; seeking help and advocacy and challenging and resisting dementia‐unfriendly practices; maintaining and adapting habitual spatial practices and being determined to ‘*get ou* *t*’; and employing similar emotional coping strategies for the pandemic and dementia.

**Conclusions:**

Support for people with dementia, especially during public health crises when carers and services are under pressure, should involve utilising existing capacities, appropriately supporting the acquisition of new knowledge and skills, ‘safety‐netting’ through the availability of a named professional, advocacy and support and use of ‘check‐in calls’ and creating supportive social and environmental circumstances for people with dementia to sustain their own well‐being.

## Key points

- Research conducted directly with people with dementia, exploring their lived experiences, is helpful for understanding the ways in which they respond as *‘active social agents’* to challenges in their lives.
- People recently diagnosed with dementia reported a range of challenges and negative experiences associated with the Covid‐19 pandemic including loneliness and loss of social routines; difficulties in accessing and trusting health services; dementia‐unfriendly practices and challenges ‘*getting out*’ into the physical neighbourhood.
- They responded to challenges by maintaining and extending social networks; learning new skills; supporting others and engaging in reciprocal exchange, including with peers; seeking help and advocacy and challenging and resisting dementia‐unfriendly practices; maintaining and adapting habitual spatial practices; and employing a range of emotional coping strategies.
- Support for people with dementia during public health crises should aim to create social and environmental circumstances that enable people with dementia to help sustain their own well‐being.

## 1 BACKGROUND AND OBJECTIVES

The Covid‐19 pandemic has presented widespread challenges for people's physical and mental health, well‐being and quality of life.^1^ From the outset, there was concern that impacts would be particularly egregious for people with dementia.^2^ Reasons included concerns that they would find it harder to comply with protective measures, lack access to necessary support, not cope well with disrupted routines and be deprived of important social and cognitive stimulation. Since then, various international studies have described wide‐ranging negative impacts for people with dementia, including discontinuation of care services, social isolation, cognitive decline, and behavioural and psychological symptoms such as apathy, impaired sleep, anxiety, depression, aggression, and repetitive behaviours.^3^, ^4^, ^5^, ^6^

Identifying negative outcomes is important, but academic and policy debate framed solely around negative impacts risks inadvertently reproducing a discourse that positions people with dementia as entirely passive.^7^, ^8^, ^9^ This is compounded by a lack of qualitative research conducted directly with people with dementia to explore their lived experiences of the pandemic.^10^ That which exists presents a more nuanced picture. Talbot and Briggs,^11^ for example, found that, during the pandemic, people with dementia in England experienced *‘a shrinking world.’* This is a concept first proposed by Duggan et al.^12^ who argued that the symptoms and impacts of dementia, including declining memory, disorientation, anxiety, and reduced confidence, can create a negative cycle, causing people with dementia to progressively reduce the frequency and scope of their outdoor activity. In the context of the pandemic, Talbot and Briggs^11^ found that people with dementia experienced similar difficulties resuming their usual routines following the initial national lockdown in March/April 2020, with potential consequences for their longer‐term independence and well‐being. However, this was experienced ambivalently by participants; on one hand, they missed social contact, cognitive stimulation and meaningful activities in the outside world but, on the other, reported feeling safe in their ‘*lockdown bubble’,* appreciated respite from having to interact with others and valued having time to learn new skills or resume neglected hobbies. Some quantitative research also suggests a more equivocal picture. For example, longitudinal quantitative research undertaken for the DETERMIND study with people newly diagnosed with dementia in England^13^ found that quality of life, measured before and during the pandemic using the dementia‐specific DEMQOL system,^14^ had not statistically‐significantly changed, regardless of whether quality of life was self‐ or proxy‐rated, or whether people with dementia lived alone or with a carer.^15^

A range of studies, from different disciplinary perspectives, provides potentially helpful context for these mixed findings. Key is research by Bartlett and O’Connor,^7^ who make the case for understanding people with dementia as *‘active social agents’* and ‘*citizens’*, with multiple social roles and statuses, a diversity of aptitudes and personal circumstances, and the ability to act for themselves and others in society. Their work forms part of a growing literature exploring people with dementia's ability to adapt, cope, seek meaning and maintain quality of life in the face of dementia‐related challenges.^16^ This wider literature includes research that considers how people with dementia interact with their local community and neighbourhood. Notably, Ward et al.^17^ argue that Duggan et al.‘s^12^ ‘*shrinking world*’ concept provides a *‘partial and unhelpfully negative picture of neighbourhood life that reinforces ideas of the passivity of people with dementia’*. Based on qualitative research with 67 people with dementia conducted before the pandemic (2014–2019), Ward et al. found that people with dementia commonly *‘push back’,* including by trying to protect loved ones from the demands of their condition, engaging in reciprocal exchange, building new social networks, challenging or resisting negative stereotypes through visibility in public spaces or through social contribution, adapting how they traverse their neighbourhood, seeking to maintain habitual spatial practices, and by actively drawing upon neighbourhood resources. Bartlett and O’Connor^7^ and Ward et al.^17^ emphasise how socio‐political‐environmental contexts support or limit people with dementia's attempts to cope, adapt and exercise agency. A systematic meta‐synthesis of evidence on coping in dementia by Bjørklof et al.^18^ considers similar issues from a socio‐psychological perspective. They identified four main psychological strategies (acceptance, keeping going as usual, adapting and adjusting to the new situation, and avoidance) and two resources (practical and emotional support, and humour).

In this paper, we aim to identify the challenges that people newly diagnosed with dementia perceived themselves to have faced as a result of the Covid‐19 pandemic and, drawing upon the multiple perspectives described above, texamine how they attempted to cope with and adapt to these challenges, and the factors that helped or hindered them.

## 2 RESEARCH DESIGN AND METHODS

### 2.1 Design

We adopted a qualitative research design using in‐depth interviews for their ability to provide rich, descriptive data about people's lived experiences and interpretations of events. Bartlett and O’Conner's^7^ concept of people with dementia as ‘*active social agents*’, Ward et al.‘s^17^ concept of people with dementia ‘*pushing back*’ and socio‐psychological concepts of emotional coping in dementia informed the research aims and design by determining phenomena of interest for data collection and analysis. In particular, these concepts focused the research not just on challenges and potentially negative impacts, but also on how people newly‐diagnosed with dementia coped and adapted and the factors that helped or hindered them. To allow for new insights and novel findings, we adopted flexible, in‐depth data collection methods and a data‐led, thematic approach to analysis.

### 2.2 Participants

Twenty‐one people newly‐diagnosed with dementia (7–14 months prior to interview) were purposively selected for maximum variation from a sample of 93 people participating in the DETERMIND‐C19 study. The DETERMIND‐C19 sample was nested within a cohort of 266 people with dementia recruited from memory clinics in three geographical areas in England (Gateshead, South London and Sussex) participating in the DETERMIND (DETERMINants of quality of life, care and costs, and consequences of inequalities in people with Dementia and their carers) research programme.^13^ Wide‐ranging baseline information to assist in sampling was available. Participants were selected across geographical areas and on the basis of gender, age, ethnicity, educational attainment, relative deprivation (Index of Multiple Deprivation) and quality of life score (DEMQOL^14^;). We also included participants living alone, including those with no identifiable carer, whose perspectives are frequently excluded from research studies. Participants meeting the sampling criteria were approached by DETERMIND‐C19 researchers and invited to take part. They were told what participation would involve, how their data would be handled, that their participation was entirely voluntary and given the opportunity to ask questions. If interested, an interview was arranged. If they declined, another member of the cohort with similar socio‐demographic characteristics was selected. The achieved sample provided a high level of variation across all participant characteristics except for ethnicity (majority were White British) and sexual orientation (all identified as heterosexual). Table 1 describes the achieved sample.

**TABLE 1:** Participant characteristics

Characteristics	*n* (*N* = 21)
Age	
<65	1
65–74	6
75–84	10
≥85	4
Gender	
Female	12
Male	9
Heterosexual	21
Relationship	
Married	10
Widowed	8
Divorced/separated	3
Living situation	
Alone	8
With carer	12
Care home	1
Location	
South London	6
Sussex	13
Gateshead	2
Area	
Urban	12
Rural	9
DEMQOL baseline (follow up)^a^	
≤70	2 (1)
71–80	1 (2)
81–90	3 (5)
91–100	7 (7)
≥101	8 (6)
DEMQOL change during pandemic	
Maintained	2
Decreased	7
Increased	12
Type of dementia	
Alzheimers disease	15
Vascular dementia	1
Mixed	2
Not known or other	3
MMSE score at diagnosis (range 7–14 months, average 10 months, prior to interview)^b^	
Minimal 26–30	14
Mild 20–25	7
Moderate 10–19	0
Severe 0–9	0
Education	
No qualification	5
Level 3 Upper secondary (GCSE)	6
Level 4: Post‐secondary non‐tertiary (A‐level)	3
Level 5: Short‐cycle tertiary (occupational)	3
Level 6: Bachelors or equivalent	2
Level 7 or 8: Masters or Doctorate or equivalent	1
Not known	1
Index of multiple deprivation (IMD) quintile	
1 (most deprived)	1
2	4
3	6
4	8
5 (most affluent)	2
Ethnicity^c^	
White British	18
Indian	1
Mixed ethnic background	1
Other ethnic group	1

^a^ Baseline scores were obtained prior to the Covid‐19 pandemic. Follow up scores were taken during Covid‐19 at the time of the interview.

^b^ Face‐to‐face measures such as MMSE scores could not be measured at the time of interview because of Covid‐19.

^c^ International Standard Classification of Education.

### 2.3 Data collection

Because of Covid‐19 restrictions, interviews were conducted by telephone or video‐conferencing platform (e.g. Zoom). They took place between November 2020 and January 2021, at a time to suit the participant. Participants were able to attend with a carer or companion, if they wished. They were also able to undertake the interview in multiple shorter sessions, although this option was not taken up. Participants were informed in advance and, as appropriate, during the interview that they could take a break or end the interview at any time. Participants reported a range of experiences but it is possible that those in the most challenging circumstances felt unable to participate. Those less able to communicate remotely, for example, because of hearing impairments or communication difficulties, may have felt similarly unable to participate.

In interviews, participants were asked broadly about their experiences during the pandemic. A topic guide (see supplementary material) was developed by the lead author (JD) and two researchers (BH, KG), in consultation with the wider research team. It included three open‐ended questions designed to initiate conversation about i) the current period and period since the March/April 2020 national lockdown ii) the March/April 2020 lockdown itself, and iii) expectations and hopes for the future. These periods were drawn deliberately broadly since participants with dementia were likely to have particular difficulty recalling details of changing restrictions over time or linking these to their personal experiences. Regulations in England during this period included the initial national lockdown (March/April 2020), incremental easing of restrictions over Summer 2020 followed by tightened restrictions and another national lockdown in November 2020, a tiered system of restrictions in December 2020 and a further national lockdown in January 2021. Full details of the English Government's Covid‐19 regulations are available online from the House of Commons Library.^19^ For each of the three open‐ended questions, further questions and prompts were provided to guide interviewers and ensure coverage of key topics. The topic guide, however, was designed to be used flexibly, allowing participants to focus on issues that were of most salience to them and to discuss topics in naturalistic, organic ways. This flexible approach was adopted to fully ground findings in the lived experiences, perspectives and priorities of participants. Ethically, it also allowed participants to guide conversation towards or away from particular topics as they preferred, given the greater difficulty for interviewers in anticipating and identifying participant distress remotely. The lead author (JD) and two researchers (BH, KG) conducted the interviews. To ensure a consistent approach, they met regularly throughout the fieldwork. The interviews lasted, on average, 55 min (one lasted 10 min, and the remainder 40–110 min). Interviews were audio‐recorded and transcribed verbatim.

### 2.4 Data analysis

Data management and analysis were conducted using Framework, a theoretically and methodologically flexible tool suitable for collaboration in large research teams.^20^, ^21^ Within this, we undertook thematic analysis using Braun and Clarke's^22^ six steps: familiarization, coding, generating themes, reviewing themes, defining and naming themes, and writing up. An initial Framework was developed by the lead author (JD), drawing on the topic guide and familiarisation with transcripts. The lead author (JD) and two researchers (BH, KG) then tested the Framework by ‘charting’ several transcripts, leading to further refinements. The Framework was then discussed with a multi‐site team of 11 researchers and additional refinements undertaken. The final set of Framework headings (codes) were descriptive and covered current situation and circumstances; physical and mental health; daily activities; personal activities; relationship with carer; support from friends and neighbours; health, care and community‐based services; attitudes to the Covid‐19 pandemic restrictions; and hopes and expectations for the future. Nine researchers comprehensively charted remaining transcripts into the Framework, with scope for them to record their own observations concerning potential themes and how specific data addressed the research aims. The completed Framework was then shared so team members could familiarise themselves with the dataset and an online meeting was convened to identify and discuss key themes. Using multiple researchers, in this way, can enhance trustworthiness and support reflexivity by allowing for assumptions to be challenged and for a range of alternative interpretations to be identified.^23^ ‘Central charts’,^21^ exploring themes and relationships in the data, were then developed by the lead author within the context of ongoing team discussions. These secondary charts re‐presented data from different areas of the original Framework chart under new and evolving thematic headings.

#### 2.4.1 Ethical permissions

Ethics approvals for the DETERMIND and DETERMIND‐C19 studies were obtained from the NHS Brighton and Sussex Research Ethics Committee [REC 19/LO/0528].

## 3 FINDINGS

We first describe the specific challenges that participants faced as a result of the Covid‐19 pandemic (3.1) and then go on to describe the different ways in which participants responded to these challenges (3.2).

### 3.1 Challenges faced by participants during the Covid‐19 pandemic

The key challenges identified were:

- reduced social contact, increased loneliness, loss of social routines
- difficulties in accessing and trusting health services
- dementia‐unfriendly practices in public spaces
- disparate experiences of being able to ‘get out’ into the physical neighbourhood.

#### 3.1.1 Reduced social contact, increased loneliness, loss of social routines

Participants generally limited social contact outside their household or residential setting in response to national lockdowns and social distancing and travel restrictions, and from fear of contracting or transmitting the virus. Meeting with family members or friends was significantly reduced or restricted to one person in a ‘support bubble.’ Some participants shielded because they or someone in their household was clinically vulnerable, or they were unable to see family members or friends who were shielding. Occasionally, participants welcomed reduced social contact, for example, to invest time in hobbies. However, more commonly, loss of contact with valued friends or family members was described as *“upsetting”* or *“depressing”*. In one case, a participant living in a care home reported significantly reduced social contact, with loss of visits, extensive restrictions on leaving the care home, less interaction with over‐stretched staff, social distancing measures within the home and self‐isolation following regular hospital visits, leading to depression requiring treatment.

Some reported being already socially‐isolated before the pandemic; contributing factors included living alone, comorbid health conditions, moving to a new area, poor family relationships and family protectiveness. For some of these individuals, Covid‐19 pandemic restrictions had consequently made little difference.

The lock‐down has had an effect on my life, but marginally. I was already pretty locked down before.(Male, age 84)

However, more commonly, Covid‐19 restrictions compounded existing loneliness.

I've learned how to be lonely but, you know, that wasn't just through Covid, it was before, but much more intensified now.(Female, age 80)

It's the way we could connect, you know, going to the shops, talking to the shopkeeper, buying a newspaper. That's all gone for us, you know, as people who have minimal contact.(Female, age 82)

The closure of organised groups and activities was also discussed. These gave opportunities for socialising and physical and mental stimulation, and could provide participants with structure, purpose, identity and meaning.

What I was saying about the not wanting to get up. If we didn't have this pandemic on, I'd have been going to the [name] Club every Tuesday, sorted. Right, get up, get my breakfast, go off to the [name] Club.(Male, age 66)

This loss was keenly felt where participants had attended groups several times a week, with one describing it as *“the worst thing that happened to me”* (Female, age 79) and another commenting, *“that's why I found it so lonely, I'd been so used to going out.”* (Female, age 85). Some participants had also been considering joining a dementia group but, because of the pandemic, had been unable to do so.

#### 3.1.2 Difficulties in accessing and trusting health services

Participants described health services being delivered remotely. Some valued not having to visit a hospital or GP surgery while there was perceived to be a high risk of contracting Covid‐19. However, remote health consultations were perceived as more difficult and of lower quality, with participants sometimes feeling that doctors had misdiagnosed or failed to resolve recurrent illnesses. Similarly, online counselling was experienced as confusing. Where usual GP home visits ceased, participants could be reluctant, or feel unable, to visit the surgery and sometimes reported feeling abandoned. Another participant had lost trust in health providers, referencing a perceived public discourse framing older and disabled people as *“disposable”* (Female, aged 71). Although some described positive experiences of hospital care, others declined hospital appointments for fear of contracting, or being treated alongside patients with, Covid‐19.

So, it’s a bit frightening. I’ve got a chest infection, and that’s a cough, so they’re bound to try Covid first, aren’t they?(Female, age 80)

#### 3.1.3 Dementia unfriendly practices in public spaces

Dementia‐unfriendly practices were described, in which staff in retail and public spaces implemented Covid‐19 restrictions inflexibly or impatiently. For example, participants sometimes forgot their masks on public transport, eliciting inconsistent and sometimes unsupportive responses from staff.

I've got bad feet. I got on a bus, and the little bugger, the driver, made me go upstairs, because I'd forgot my mask.(Female, age 85)

These experiences occurred even when participants adopted measures designed to ensure they are identified as someone with dementia and given extra support. For example, one participant described his poor experience of service in a local bank while he was wearing an Alzheimer's badge.

'No, you can't come in.’ I'm trying to explain, you know, and she could see I had the Alzheimer's badge on. There was no helper, so I just felt very put off, because I felt frightened with it.(Male, age 66)

#### 3.1.4 Disparate experiences of being able to ‘get out’ into the physical neighbourhood

Participants spoke widely of the importance of *“getting out*” during the pandemic, including for exercise, fresh air, a sense of freedom and being in nature. Rural areas provided multiple options for local walks, had lower community levels of infection and unwanted close contact with others was more easily avoided. Those in rural areas also more commonly reported supportive relations with neighbours, but sometimes also felt isolated given disrupted public transport services and fewer opportunities to spend time in local town centres. In contrast, those in urban areas worried more about contracting Covid‐19 and walks out were generally more limited, commonly *“around the block”* or in local parks and green spaces. They sometimes described feelings of being *“cooped up”* or *“incarcerated,”* occasionally comparing the pandemic with wartime. Some also reported getting less exercise, which could exacerbate existing health problems.

I do suffer with COPD, that could be affecting that a bit. During this period of time I haven't been exercising properly because I haven't been out and about.(Male, age 78).

However, people's ability to ‘*get out*’ was influenced also by a range of non‐pandemic‐related factors, including limiting comorbid conditions and mobility impairments.

### 3.2 Participants responded actively to these challenges

While participants experienced challenges and negative impacts, they also described multiple ways in which they resisted, coped with and adapted to challenges. These were:

- maintaining and extending social networks, making the most of ‘nodding acquaintances'
- learning new skills, for communication and hobbies
- supporting others, engaging in reciprocal exchange and valuing connection with peers
- seeking help and advocacy, challenging and resisting dementia‐unfriendly practices
- maintaining and adapting habitual spatial practices, being determined to ‘*get out*’
- employing similar emotional coping strategies for the pandemic and dementia.

#### 3.2.1 Maintaining and extending social networks, making the most of ‘*nodding acquaintances*’

Participants were sometimes motivated to sustain and extend social connections despite pandemic restrictions by an awareness that they were in their final years.

We have not got that much time left, and we don't particularly want to be stuck indoors, on our own.(Female, aged 71)

They formed ‘support bubbles’, usually with family members, and used the telephone or digital platforms to stay in touch with friends and family. Participants described initiating or actively seeking contact with family members when they felt they needed it. For example, one participant requested more telephone contact from her daughter in response to growing feelings of loneliness and depression.

I just suddenly started feeling it wasn't worth living. And, well, you know, I just got a bit fed up, and I texted my daughter, and she suddenly realised that I needed more contact. She phones every day now.(Female, age 85)

Some found that greater reliance on remote communication during the pandemic actually increased levels of social contact, particularly with grandchildren. One participant, for example, regularly listened to her grandchildren read to her over a digital platform, which she had not done previously. Several male participants continued face‐to‐face contact with valued friends, justifying this with references to *“herd immunity”* and feeling it was okay to see *“regular friends.”* Exceptionally, participants even made new friends. For example, one participant, previously quite socially isolated, had befriended a group of immediate neighbours, who she now regularly met with in a shared garden space. Those living alone with limited social networks often sought out opportunities for safely‐distanced forms of social contact, ranging from looking out of the window at people passing to seeing people *“out‐and‐about”* and exchanging casual greetings from afar.

You've got to take advantage of that walk out. What we call it is nodding acquaintances, you're walking on and you might see someone every other week, you don't know their name. ‘Morning,’ ‘Morning.’ That kind of interaction.(Male, age 66)

Organised groups and activities that had closed during the pandemic sometimes offered video‐conference (e.g. Zoom) or telephone‐based alternatives. Some regularly attended these and found them helpful, with one participant commenting, *“it's interaction, it's seeing somebody.”* (Male, age 66). However, without a shared activity, some found there was nothing to talk about. Another participant, living alone, found that listening to others on a group call talking about, and being supported by, their families only intensified her feelings of loneliness. Those who chose not to attend remote groups, however, sometimes remained in touch with individual friends from previously‐attended groups. At the time of interviewing, some religious services and dementia groups were tentatively re‐opening, commonly with less frequent meetings, fewer participants and social distancing. While not all participants were confident to do so, some had resumed their attendance.

#### 3.2.2 Learning new skills, for communication and engaging in hobbies

Use of digital communications was important for many in mitigating the loss of face‐to‐face social contact. Participants commonly developed their IT skills to facilitate this, including by accessing training through the local library, using information on the Internet or with the, sometimes remote, help of friends and family.

I've used it a bit (virtual platforms), much more than I would have done in the old days because I'd have just gone and seen them, or they come and see me, but we've had to go down these other routes, and it's amazing how versatile these systems are, and what you can do with them(Male, aged 75)

However, others struggled to use digital communications during the pandemic.

They [members of an organised online group] seem more au fait with all the computer systems, and I feel like saying, ‘Oh, am I an idiot or something?’ I get so frustrated that I cry.(Female, age 68)

Some actively sought assistance from relatives but this was not always willingly or patiently given. Some participants, therefore, relied on telephone contact for connecting with family and friends.

Participants commonly also developed or learnt new skills to engage in hobbies, including gardening, cooking, reading, music, keep‐fit and woodwork. Participants valued these activities for their intrinsic satisfactions and benefits but also described them as effective ways of coping with the pandemic, including by providing useful distraction and escapism. Male participants, in particular, also reported gaining a sense of self‐worth, confidence and social contribution; for example, by baking bread for their family, selling woodwork through local fairs or playing piano for neighbours to listen to.

#### 3.2.3 Supporting others, engaging in reciprocal exchange and valuing connection with peers

Participants were not just passive recipients of social and practical support but also providers. Some had taken on support roles during the pandemic. For example, one participant regularly bought groceries for her daughter who had lost her job because of the pandemic, while another adapted his routine for his co‐resident daughter who was working from home. Others offered, primarily emotional (given the difficulties of in‐person contact), support to relatives experiencing ill‐health, including, one participant's sister who was undergoing major surgery and another participant's brother who had received a terminal diagnosis.

Many participants particularly valued the peer relationships they found in organised dementia groups, referring positively to being with others who had *“been through the same thing”* (Female, age 85) and *“meeting all the same people every time”* (Female, aged 85). Two male participants described the satisfaction they got from supporting and advising newer members. Although participants had not be during the pandemic, some had remained in touch with individual friends from their groups or prioritised returning to these groups as soon as possible. Others saw special value in attending online dementia groups and offering support to peers, at a time when there was less support from formal services.

So, all I can say is what I get from it and what I hope I am able to give to others in my condition. I've seen new ones now come along, early stages in their condition. I'm like well over twelve months in mine, these are only just like months, learning still, being told by a phone call [because of the pandemic] that they've been diagnosed with Alzheimer's.(Male, aged 66)

Occasionally, however, participants appreciated the pandemic restrictions freeing them of unwelcome expectations and responsibilities. One participant, for example, who had previously worked in a caring profession and had been for her granddaughter, was pleased to withdraw from caring for others.

For me this is nice and I…, not retreat, but feel I've done my bit over the years, you know … I'm not doing things for other people, you know. So, it's all the energy is towards me.(Female, age 76).

#### 3.2.4 Seeking help and advocacy, challenging and resisting dementia‐unfriendly practices

Participants generally had limited contact with dementia services but appreciated having a named professional from their memory service to call if needed. Some found this especially reassuring given a perception of a negative public discourse concerning the value of older and disabled people. In another case, a participant engaged an advocate from a local dementia organisation following an experience of dementia un‐friendly service in a high street setting. The advocate liaised with the service‐provider and secured a commitment to more dementia‐friendly practices in future.

Participants were also active about asking for, accepting and agreeing to other types of help and support. One participant, for example, agreed to counselling for depression. He was motivated and hopeful about undertaking this, despite having no previous experience of counselling and poor initial experiences of undertaking counselling online. Participants also described a wide range of help‐seeking from family and friends, including asking for more frequent contact, lifts, help with errands and shopping, and support to use digital communications technologies.

#### 3.2.5 Maintaining and adapting habitual spatial practices, being determined to ‘*get out*’

Participants commonly expressed considerable determination to ‘get out’ into their local neighbourhoods, making comments such as, *“no‐one's going to keep me in”* (Female, age 85) and *“I am frightened of it, but it doesn't stop me from going out.”* (Male, age 66). This was consistent with many similar expressions of independence. For example, one participant commented, *“I feel like saying to them, ‘I've not lost it yet, you know. Don't wrap me up in cotton wool just yet.’”* (Female, age 71) and another commented, *“I suppose I grew up to be quite stubborn and independent, and so I just got on with it.”* (Female, age 85). Only exceptionally did participants find it difficult to resume previous routines following the national lockdown. For example, one described herself as *“scared of going out”* (Female, age 86) and another noted, *“the longer you don't go out, the more you don't want to”* (Female, age 80). However, even those who had found it difficult to re‐integrate initially, sometimes made an effort to go out more later on, with one participant reflecting, *“I should have made myself go out. I should have persevered and gone out,”* (Female, aged 85).

When going out into their local neighbourhoods, participants commonly tried to keep to their usual routines but were also able to adapt their behaviours. In urban areas, for example, participants avoided inadvertent close contact with others by going out early in the morning, avoiding crowded parks or using local shops instead of large supermarkets accessible only by public transport. In one case, a participant described going out walking in the evening during the initial national lockdown so that, *“he* [Boris Johnson] *won't know about it.”* Participants also navigated disrupted public transport services, hired taxis or asked relatives and friends for lifts to access shops and medical appointments. In one case, a participant, unable to attend regular nurse appointments for an ulcerated leg because of disrupted transport services, resorted to using supplies from the local chemist and dressing it herself.

#### 3.2.6 Emotional coping, employing similar strategies for the pandemic and dementia

Participants commonly adopted similar emotional coping strategies for dealing with the pandemic as they did for their dementia. Denial or minimising of dementia by, for example, expressing doubt over their diagnosis, was a coping strategy evident in multiple accounts and was sometimes extended to the pandemic. One participant, for example, said of his dementia, *“as far as I'm concerned, it's not there, it doesn't bother me”* and of the pandemic, *“it doesn't interest me, you know, if I took an interest in it, yes, but I don't take an interest in it, so it doesn't bother me”* (Male, age 73). More commonly, participants coped with both the pandemic and their dementia by adopting an attitude of equanimity, acceptance or stoicism.

Well, you can't do anything about it [the pandemic], and I can't do anything about my dementia, you just learn to live with it.(Female, age 72)

Perspective‐taking and gratitude were also common coping strategies. Participants spoke, for example, of *“being content in what I've got, knowing there's a lot of people worse off”* (Male, age 66). Participants also employed positive framing. For example, a participant with a persistent comorbid condition commented on not receiving a GP home visit, *“I'm probably better than a lot of people, they probably go to people that are really ill”* (Female, age 80) and another participant reflected on a lack of family contact, saying the reason for this was, “*I think they think I'm quite capable”* (Female, age 72). Participants also attempted to maintain a sense of normalcy.

There's nothing I can do about the virus going around so I live my life as, you know, normal, as normal as I can.(Female, age 85)

There were, however, limits to participants' ability to emotionally cope and resilience could be eroded over time.

At the beginning of the lock‐down, I was okay, then I just made myself overcome things, at the beginning, and gradually it got worse and went downhill.(Male, age 78).

## 4 DISCUSSION AND IMPLICATIONS

People with recently diagnosed dementia in our study described a range of challenges and negative experiences associated with the Covid‐19 pandemic and its restrictions. Many of these challenges were shared with the wider population but participants' experiences were often shaped by dementia‐specific factors and some challenges, such as experiences of stigma, dementia‐unfriendly services or closure of dementia support groups, were dementia‐specific. Rather than being passive in the face of these challenges, we found that participants showed considerable creative resilience and active coping. In terms of the literature and concepts referenced in our introduction, they were *‘active social agents’*,^7^ *‘pushed back’* in multiple ways^17^ and adopted a wide variety of emotional coping strategies.^18^

We maintain that it is important not just to understand the challenges that people with dementia faced during the pandemic but also the ways in which they acted for themselves, and others, and the facilitators and barriers they encountered in doing so. Applying this wider lens can help to avoid reproducing unhelpful and stigmatising discourses that position people with dementia as a homogenous group, lacking in agency. It is also helpful for identifying potentially useful ways of supporting people with dementia to sustain their own well‐being and quality of life, particularly in the context of public health emergencies when usual support from carers and formal services may be less available.

Our focus on the experiences of those recently diagnosed is also valuable. Although popular depictions of people with dementia tend to involve those in later stages of illness,^24^ at any time, around 55% of people with dementia have mild symptoms and 32%, moderate symptoms.^25^ Our study also includes people with dementia who live alone, a group often excluded from research samples.^26^ Available estimates in England and the United States suggest that as many as a third of people with dementia living in the community live alone,^27^, ^28^, ^29^ while in Germany, this proportion may be as many as a half.^26^ Many of these individuals will have no identifiable carer.^26^, ^29^ Those living alone are more likely to have mild to moderate dementia symptoms^29^ but are also more likely to experience social isolation, poverty, poorer access to health services and unmet needs.^26^, ^29^

The main pandemic‐related challenges experienced by participants in our study were reduced social contact, loneliness and loss of social routines; difficulties in accessing and trusting health services; dementia un‐friendly practices in public spaces; and disparate experiences of being able to ‘*get out*’ into the physical neighbourhood. In response to these challenges, we found that people with dementia actively sought to maintain their existing social networks and create new opportunities for social contact; learnt new skills; supported others and engaged in reciprocal exchange; challenged dementia‐unfriendly practices and engaged in help‐seeking; maintained and adapted their habitual spatial practices and showed considerable determination to ‘*get out*’; and adopted a wide range of emotional coping strategies. In our discussion, we consider these different ways of coping and adapting and how they might be supported, in the context of public health emergencies and potentially more generally, in dementia policy and practice.

### 4.1 Maintaining social networks and creating new opportunities for social contact

Many participants attempted to sustain, and sometimes extend, their social networks. As well as staying in touch with family and friends using the telephone and digital means, some maintained in‐person contact with people outside their household, whether in a ‘support bubble,’ meeting with friends outdoors as regulations permitted or, exceptionally, by disregarding social distancing regulations. However, those living alone, in a care home and/or with a comorbid health condition were particularly susceptible to becoming more socially isolated. Those who were already socially isolated were sometimes less affected by social distancing measures. More commonly, however, loneliness worsened with the loss of already low levels of social contact. Prior to the pandemic, many participants had relied on organised groups and activities for their social contact. Remote alternatives were generally available, were valuable and worked well for some. However, they did not work well for everyone. One‐to‐one phone calls from organisers to keep in touch with those uncomfortable or unable to join online groups could provide ‘safety‐netting’ support for those most at risk of social isolation. A study with 11 people with dementia and 11 carers conducted during the pandemic that found ‘check‐in calls’ of various types were widely valued.^30^

Those reliant on casual socializing within the community attempted, during the pandemic, to substitute this with socially‐distanced interactions such as exchanging greetings at a distance or watching people passing by. These findings reflect Ward et al.‘s^17^ observation that people with dementia act to repopulate depleted social networks, as well as other research emphasising the importance of socially‐distanced interactions for older people, including with dementia, experiencing loneliness.^31^, ^32^, ^33^, ^34^ The findings also lend support to wider research emphasising the importance of supporting people with dementia to sustain contact with wider secondary and tertiary networks of friends, neighbours and casual acquaintances,^7^, ^17^ especially where they have fragile social networks or live alone.^33^, ^35^ Our findings suggest that these wider social networks are of even greater importance when, as during the Covid‐19 pandemic, carers and voluntary and civic institutions are under strain. During public health crises, these relationships may be supported through, for example, supporting local mutual aid and community‐based groups to effectively engage people with dementia, including, in socially‐distanced ways.

### 4.2 Learning new skills

We know from existing literature that, for those with dementia, reduced face‐to‐face contact may not be easily substituted with digitally‐based communications.^36^ While some participants did struggle to use digital communications technologies, many employed them successfully, often learning new skills for this, including by accessing local training opportunities. In order to limit social isolation and digital exclusion at a time when society is increasingly dependent on digital communications technologies for social connectedness,^37^ it is important that misplaced assumptions about people with dementia being unable to learn new skills does not inhibit potentially helpful developments in this area, for example, the provision of dementia‐specific support or easy‐to‐use digital interfaces. Such resources should be co‐produced with people with dementia.^38^

Participants in our study also developed new skills to support hobbies. Kitwood^39^ identified participation in occupations as one of the main psychological needs of people with dementia and our research found that, during the Covid‐19 pandemic, engagement in hobbies could help substitute, in part, for reduced social contact and the closure of organised groups. Aside from their inherent pleasures and benefits, hobbies could provide helpful distraction, avert boredom and enhance self‐esteem. These findings align with those of Talbot and Briggs,^11^ who found people with dementia valuing time for hobbies during England's initial national lockdown, and those of Bartlett and O’Connor,^7^ who found that people with dementia can undertake new, not just maintain existing, activities.^7^ In common with several existing studies, we also found that men were most likely to emphasise the value of their hobbies and activities to others.^40^, ^41^ Engagement in pastimes and hobbies could be promoted and supported as part of a living well approach, particularly in earlier stages of dementia, potentially generating resources that would be valuable in future public health crises.

### 4.3 Supporting others, engaging in reciprocal exchange and connecting with peers

Reflecting Bartlett and O’Connor's^7^ concept of people with dementia having multiple social statuses and roles, participants sometimes had responsibilities to others. Support was often offered willingly and sometimes reciprocally, although participants were sometimes grateful for the opportunity the pandemic gave to limit their commitments to others. The diversity of people's social relationships and responsibilities emphasises the importance of flexible and personalised support. In common with Ward et al.^17^ we found participants especially valued the camaraderie, giving and receiving of peer support and sharing of dementia‐specific knowledge and know‐how in dementia support groups. Some participants had been contemplating joining such groups as the pandemic started, but had not yet had the opportunity. Following the easing of restrictions, resumption of in‐person dementia groups should be prioritised, previous attendees supported to return and groups promoted to those newly diagnosed.

### 4.4 Challenging dementia‐unfriendly practices and help‐seeking

We, like Talbot and Briggs,^11^ identified dementia‐unfriendly practices. These included undifferentiated and officious application of social distancing regulations in public spaces. Participants often resisted or challenged these practices, sometimes actively seeking advocacy and support to help them. Health services were also delivered in dementia‐unfriendly ways, with people with dementia commonly left to negotiate reconfigured health care systems unsupported, sometimes resulting in foregone healthcare and potentially poorly‐managed comorbid conditions. Some described feeling that health providers did not care about them. Others also perceived there to be a stigmatising public discourse about the value of older and chronically‐ill people.

In the context of a public health crisis, when most at risk of being eroded, dementia‐friendly practices should not just be maintained but, as far as possible, strengthened. This should be augmented with available advocacy and support, allowing people with dementia to take the lead in defining and challenging such practices. While acknowledging pressures upon health services during the pandemic, people with dementia also appeared to require more support to access the health care they needed. ‘Safety‐netting’ support from dementia care services during the pandemic was especially valued. While rarely called upon, participants gained considerable reassurance from having a trusted professional to contact in case of problems. While, in England, a named care co‐ordinator is recommended by the National Institute for Health and Care Excellence,^42^ and much valued by people with dementia,^43^ this is currently still far from the norm. Check‐in calls for those at greatest risk of foregoing healthcare may also be beneficial.^30^

### 4.5 Maintaining and adapting habitual spatial practices

We found only limited evidence to support the idea of people recently diagnosed with dementia experiencing a *‘shrinking world’*.^12^ Where people did withdraw, as in Talbot and Briggs' study (2021), this was sometimes because they took the opportunity to focus positively on their home life and hobbies. Others were limited by comorbid health conditions and mobility impairments, the need to shield or strict social distancing regulations in care homes rather than by their dementia symptoms. More commonly, we found that participants *‘pushed back,’* frequently expressing strong determination to remain engaged with their local communities and physical neighbourhoods, even in densely populated urban areas where concerns about contracting Covid‐19 were greater. The presumption that people with dementia will inevitably withdraw from public life should be challenged. Dementia‐friendly community initiatives can support continued engagement^33^; in the context of the pandemic, there was, for example, an apparent need for greater support to utilise priority shopping times. Attention should also be given to mitigating non‐dementia‐specific barriers to neighbourhood access such as those presented by health and mobility impairments, and lack of accessible public transport.

### 4.6 Developing effective emotional coping strategies

We found that participants commonly employed similar emotional coping strategies during the pandemic to those they used to adapt to their dementia diagnosis, including denial, acceptance, stoicism, perspective‐taking and positive framing. These reflected strategies identified by Bjørklof et al.^18^ in their systematic review of coping strategies in dementia. Although Bjørklof et al. identified humour as a key resource for coping with dementia, this was not discussed in relation to coping with the pandemic. Recent theories of emotional coping emphasise the importance of context and flexibility.^44^ While strategies such as denial or distraction are often considered detrimental to wellbeing,^45^ in some circumstances they may be beneficial, reducing anxiety or stress in especially challenging circumstances, for example, on receiving a dementia diagnosis or during a pandemic. Emotional coping strategies may also involve trade‐offs; denial and failing to socially distance from friends, for example, may limit social isolation but increase the risk of contracting Covid‐19, while too much positive framing may lead to accepting poor quality services or not asking for needed help. Interventions, accessible and appropriate for people with dementia, that promote adaptability and flexibility in emotional coping may be especially helpful in the context of public health crises.^46^

## 5 CONCLUSIONS

In the context of a global public health crisis, it is understandable that research and policy focus on vulnerabilities and negative outcomes. Nonetheless, as Ward et al.^17^ note, people with dementia are capable of *‘taking steps to rebuild their worlds; fostering new friendships, engaging in opportunistic sociability and endeavouring to keep places of importance reachable and accessible.’* Our research shows this to be no less true in the context of the Covid‐19 pandemic. Support for people with dementia, especially during public health crises when carers and services are under pressure, should focus on utilizing people's full capacities, supporting them in the acquisition of new knowledge and skills where helpful, ‘safety‐netting’ through the availability of a named professional, advocacy and support and use of ‘check‐in calls’, and creating supportive social and environmental circumstances for people with dementia to promote their own well‐being.

## CONFLICT OF INTEREST

We have no conflict of interest to declare.

## Supporting information

Supplementary Material

## Data Availability

Research data are not shared.
