# Supplementary material for: ‘Pushing back’: People newly diagnosed with dementia and their experiences of the Covid‐19 pandemic restrictions in England
Source: Int J Geriatr Psychiatry. 2022 Sep 2;37(10):10.1002/gps.5803. doi: 10.1002/gps.5803 (PMC9539182; doi:10.1002/gps.5803)
Supplement: Supplementary file 1 — Supplementary Material [file GPS-37-0-s001.docx]

**Topic guide (supplementary material)**

This interview is designed to identify the main challenges that participants faced in relation to the Covid-19 pandemic regulations, how they managed and responded to these challenges and what helped and hindered them. The interview is designed to be in-depth and to focus on the issues that are most salient to participants. Consequently, the topic guide is designed to be used flexibly; you do not need to delve into all of the areas in the same amount of detail and you do not need to cover them in the order outlined below.

Remember to encourage the participant to talk in detail about their experiences. Useful prompts could be:

- Why do you say that?
- Can you tell me more about that?
- Can you give me an example of that?

If participants have any recall difficulty, gently reassure them that it is not important, let them know that you can come back to that later and move to a new topic. Let participants know that they can take a break or end the interview at any time without having to give a reason.

1. Can you tell me, how are things going at the moment?
   Probe for whether this has changed in the last few months and/or since the initial lockdown (March/April 2020)?

- What is their living situation at the moment (e.g. their home and local area, who they live with)?
- What are their daily activities at the moment (within and outside of the home)? What, if anything, is helping them to maintain these activities? Are they facing any challenges in maintaining their daily activities?
- What is their relationship like with their carer at the moment (if they have one)? How are they being supported by their carer (if they have one)?
- How, if at all, are they being supported by others, such as friends and neighbours?
- How are they getting supplies such as food/medicines (e.g. online, visiting shops)? How is that going?
- What is their health like at the moment? How are they managing their health conditions? Explore to what degree their health or health care may have been affected by the Covid-19 pandemic regulations.
- Are they accessing any dementia services at the moment? If so, how are they finding them?
- Are they having much social contact at the moment? Is this with family/friends/neighbours? What form does this take? How is this being facilitated, if at all? What are the barriers?
- Are they getting out of the house much? If so, where to and for what purpose? How is that going?
- Are they clear about the rules around the easing of the lockdown and/or other changes to the Covid-19 regulations? How are they finding them? Are there any challenges?
- What has helped them most? What have been the greatest challenges?

1. Can you tell me a bit about what things were like for you during the lockdown (March/April 2020)?

- How well did they understand the Governmental restrictions associated with the lockdown and were able to follow them? Did they receive a shielding letter?
- In general, what was the lockdown like for them?
- How was their relationship with their carer (if they have one) during this period?
- How much social contact were they having with family/friends/neighbours? What form did this take? How was this managed?
- Were they making use of any services during the lockdown period (e.g. dementia services, health care services, community-based services)?
- Who, if anybody, was helping them during the lockdown (e.g. family, neighbours, professionals)?
- What helped them most during the lockdown period? What were the biggest challenges for them during the lockdown?

1. Can you tell me what you think the next few months are going to be like for you?

- Do they think much will change in their daily lives?
- Can they foresee any additional challenges?
- What do they think would help them to live better during this period?

1. Thinking about everything we have talked about today…
   - What is the biggest challenge you think you have faced during this period?
   - How well do you think you have overcome this challenge?
   - What has helped you the most in addressing this challenge?
   - What other support would you have liked during this time?
   - What advice would you give to other people who have recently been diagnosed with dementia about coping with the Covid-19 pandemic regulations and/or lock-downs?
2. Is there anything else you would like to discuss that we have not touched on?
